# Current and Emerging Therapies for Chronic Subjective Tinnitus

**DOI:** 10.3390/jcm12206555

**Published:** 2023-10-16

**Authors:** Ki Wan Park, Peter Kullar, Charvi Malhotra, Konstantina M. Stankovic

**Affiliations:** 1 Department of Otolaryngology-Head and Neck Surgery, Stanford University School of Medicine, 801 Welch Rd., Palo Alto, CA 94305, USA; 2 Department of Neurosurgery, Stanford University School of Medicine, 453 Quarry Rd., Palo Alto, CA 94305, USA; 3 Wu Tsai Neurosciences Institute, Stanford University, 290 Jane Stanford Way, Stanford, CA 94305, USA

**Keywords:** tinnitus, emerging therapeutics, electrical stimulation, neuromodulation, nerve block, CBT, EMDR

## Abstract

Importance: Chronic subjective tinnitus, the perception of sound without an external source for longer than six months, may be a greatly debilitating condition for some people, and is associated with psychiatric comorbidities and high healthcare costs. Current treatments are not beneficial for all patients and there is a large need for new therapies for tinnitus. Observations: Unlike rarer cases of objective tinnitus, chronic subjective tinnitus often has no obvious etiology and a diverse pathophysiology. In the absence of objective testing, diagnosis is heavily based on clinical assessment. Management strategies include hearing aids, sound masking, tinnitus retraining therapy, cognitive behavioral therapy, and emerging therapies including transcranial magnetic stimulation and electrical stimulation. Conclusions and relevance: Although current treatments are limited, emerging diagnostics and treatments provide promising avenues for the management of tinnitus symptoms.

## 1. Introduction

Tinnitus, derived from the Latin verb *tinnire* meaning ‘to ring’, describes a conscious perception of an auditory sensation in the absence of a corresponding external stimulation [1]. Tinnitus is an ancient phenomenon, and an early description is found in the Babylonian Talmud which provocatively describes the curse of Titus as tinnitus caused by a gnat which “pecked at his brain” [2].

Tinnitus is classified as subjective when experienced only by the individual, or, rarely, objective when the tinnitus can also be detected by others [1]. The sensation is often described as sizzling or ringing but it can be rhythmic or pulsatile in nature [1]. Tinnitus can have a sudden onset and an acute time course although more commonly the onset is gradual and follows a chronic time course [3]. Somatosensory tinnitus can be modulated by afferents from the cervical region or temporomandibular joint [4]. The most common form of tinnitus is subjective and non-pulsatile, without other known pathological processes other than hearing loss [5]. This form, which is the subject of this review, is referred to as chronic subjective idiopathic tinnitus.

Due to the lack of objective markers of tinnitus, estimations of its prevalence rely on validated patient questionnaires, which may fail to capture the true burden of tinnitus given the heterogeneity of patient experience. It is estimated that tinnitus affects more than 740 million adults globally (14%), with over 120 million people (2%) perceiving tinnitus as a major problem [6]. Severe disturbances associated with tinnitus commonly manifest as emotional distress, insomnia, reduced concentration, and cognitive dysfunction [7,8,9]. Prevalence increases with age and is similar in men and women [10,11,12]. Children and adolescents are also commonly affected, although the prevalence varies considerably in the literature (4.7 to 46%), based on a recent systemic review [13]. However, it is estimated that only a small proportion (3.1%) have problematic tinnitus based on a prospective population-based study in the United Kingdom (UK) [14]. This form of problematic chronic tinnitus has been referred to as tinnitus disorder to reflect the associated psychological and physical impacts [15].

Financially, tinnitus also places a large economic burden on healthcare systems and may lead to a significant number of disability claims. The average cost of tinnitus-related costs per patient yearly is estimated at USD 660 in the US (2014) and GBP 717 in the UK (2017) [16,17]. In 2012, an estimated USD 1.2 billion was spent on tinnitus-related compensation by the United States Department of Veterans Affairs (VA) [18].

The primary risk factor for tinnitus development is hearing loss, and additional risk factors for severe tinnitus include stress, increased age, and head injury [19,20,21]. This relationship is not linear, and many people with tinnitus have audiometrically normal hearing while many with severe hearing loss do not report tinnitus [19]. Several modifiable lifestyle factors have been associated with the development of tinnitus, including noise exposure, diet, obesity, smoking, and alcohol intake [22,23,24]. Pharmacological agents are also an important cause of tinnitus. Aminoglycosides, platinum-based chemotherapy, and salicylates are all known ototoxic agents associated with the risk of tinnitus [25]. Tinnitus is a common feature of otological and lateral skull base disorders, such as Meniere’s disease, otosclerosis, and vestibular schwannoma [10]. Tinnitus may be worsened by sleep quality and can coexist with mental health disorders including anxiety and depression [26,27]. Additionally, genetic conditions such as Williams syndrome have also been implicated in tinnitus, co-existing with hyperacusis [28]. This is thought to result from cochlear fragility and possible auditory nerve dysfunction resulting in high frequency hearing loss [28,29].

## 2. Etiology and Pathophysiology

**Figure 1:**
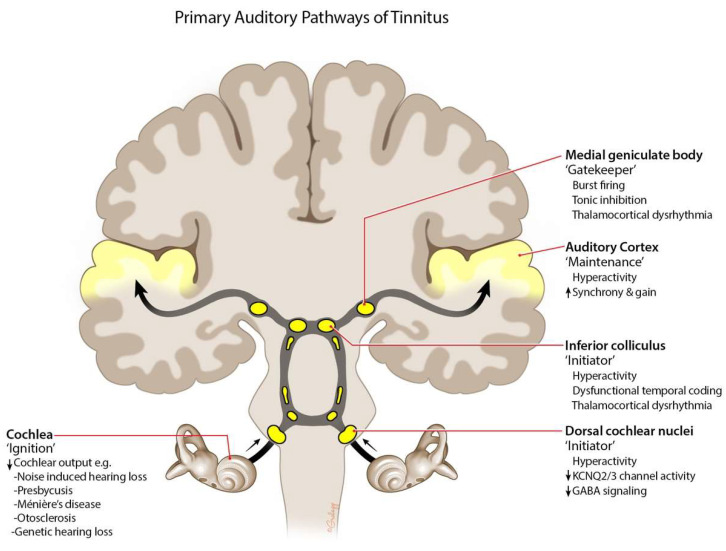
**Auditory pathophysiology mechanisms of tinnitus**. The primary pathophysiology mechanism includes reduction in cochlear output, resulting in an aberrant neuroplastic response. Abnormal dysfunctional neuronal activity in the remainder of the auditory pathway, including the ventral cochlear nucleus, inferior colliculus, medial geniculate body, and auditory cortex, is thought to be involved in tinnitus maintenance. Up arrow refers to gain, and down arrow refers to reduction. Adapted from Henton et al., 2021 [30].

There is a current consensus that the origin of subjective tinnitus is cochlear dysfunction that provokes an aberrant central neuroplastic response [30]. Jastreboff’s neurophysiological model of tinnitus proposes that cochlear damage is the ‘ignition’ event leading to altered activity in the limbic, autonomic, and reticular systems that promote chronic tinnitus (Figure 1) [31]. Cochlear damage can include loss of outer hair cells (OHCs) and inner hair cells (IHC), loss of synapses between IHC and type 1 spiral ganglion neurons (synaptopathy), or mechanical damage to hair cells’ stereocilia or the cochlea’s basilar membrane [32]. It was previously believed that tinnitus could occur without cochlear damage; however, it is now evident that this damage may occur before hearing loss becomes clinically apparent [33]. For example, tinnitus patients both with and without hearing loss have been shown to have significantly different OHC function than normal subjects (as measured with distortion product otoacoustic emission (DPOAE)) [34], suggestive of OHC loss or damage that has not yet impacted audiometric thresholds. Indeed, hearing loss was undetectable in rats treated with an ototoxic drug (styrene) until >33% of OHCs were lost [35]. Additionally, the acoustic characteristics of tinnitus perception often correspond to the region of hearing loss (i.e., high-pitched tinnitus with high-frequency hearing loss) [36].

Following cochlear damage, the according reduction in auditory nerve output is proposed to initiate a neurobiological signaling cascade resulting in hyperactivity in the central auditory system encoding for tinnitus [30]. Thus, it is possible that the loss of cochlear afferent activity liberates involuntary, internally generated percepts in the brain, similar to the neural mechanism for phantom limb pain [30]. This would explain the common clinical finding that tinnitus persists even after destruction of the auditory nerve via, for example, surgery for vestibular schwannoma [37]. This theory is further bolstered by the high rate of acquired hearing loss in tinnitus patients (~90%), the rarity of tinnitus among the congenitally deaf, and that tinnitus is often suppressed by cochlear implants which functionally replace cochlear nerve output [30,38,39]. This mechanism for tinnitus is clinically relevant because treatment at the peripheral site (e.g., sound masking at the cochlea) may not correct alterations of activity in the central auditory pathway responsible for tinnitus persistence. However, restoring cochlear input via hearing-aid-mediated sound therapy can be effective in improving patients’ subjective experience of tinnitus, and approximately 80% of patients with chronic tinnitus reported improvement in tinnitus annoyance and loudness in a questionnaire-based study in Japan [40].

Animal models of tinnitus—primarily achieved via loud sound and/or ototoxic agents following operant or reflexive conditioning to silence—have played a valuable role in understanding the pathophysiology of tinnitus [41]. Through these models, neuronal hyperactivity and hyporeactivity, neuronal gain and synchrony, and tonotopic reorganization in the brain have been associated with tinnitus symptoms [30,41]. Persistent dysfunctional neuronal activity in the ventral cochlear nucleus, inferior colliculus, medial geniculate body, and auditory cortex are thereby proposed to be responsible for tinnitus maintenance (Figure 1) [42,43,44,45]. In turn, these alterations in activity have been attributed to changes in glycinergic, GABAergic, glutaminergic, and cholinergic systems [46,47]. For example, dorsal cochlear nucleus hyperexcitability is possibly due to a reduction in potassium transport via KCNQ channels [48].

**Figure 2:**
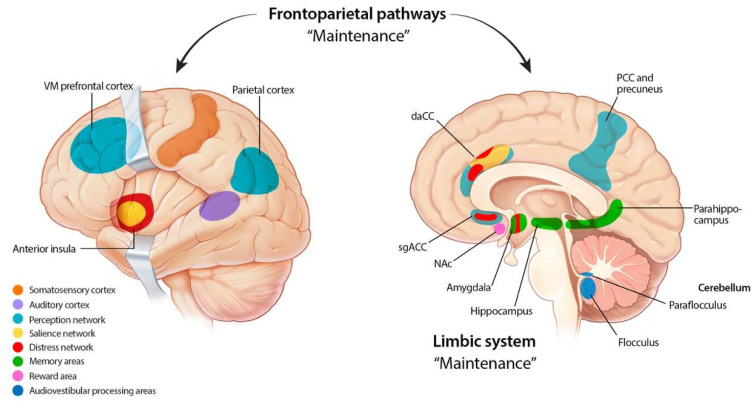
**Non-auditory pathophysiology mechanisms of tinnitus**. Non-auditory pathways play a critical role in the maintenance and affective response to tinnitus. Aberrant neuroplastic responses include the somatosensory cortex, perception networks, salience networks, distress networks, memory areas, reward areas, and audiovestibular processing areas. daCC—dorsal anterior cingulate cortex. sgACC—Subgenual anterior cingulate cortex. NAc—Nucleus accumbens. PCC—Posterior cingulate cortex. VM—Ventromedial. Adapted from Haider et al., 2018 [59].

Additionally, non-auditory pathways play a critical role in the maintenance and affective response to tinnitus (Figure 2) [49]. Correspondingly, tinnitus is known to be co-morbid with depression and anxiety [50], and intracochlear-glucocorticoid-mediated glutamate release may be a link between psychological stressors and tinnitus [51]. The frontostriatal gating theory posits that the nucleus accumbens and ventromedial prefrontal cortex are important in the affective response to tinnitus [52]. Perception of tinnitus demands attentional resource, and accordingly patients with tinnitus have been shown to have poorer selective attention on auditory tasks [8]. Correspondingly, neuroimaging studies have detected changes in the attention networks [53]. The flocculus and paraflocculus, small lobes of the cerebellum, have also been implicated in tinnitus and auditory processing [54]. Animal tinnitus models have demonstrated feedback loops between the flocculus and the auditory cortex, with cochlear damage leading to upregulation of unipolar brush cells present in the cerebellum [54,55,56]. Similarly, neuroimaging studies have detected changes in the cerebellar regions, and a study of patients undergoing cerebellopontine tumor removal have demonstrated a correlation between flocculus volumes and tinnitus severity [57,58]. However, further investigative studies are needed before definitive conclusions can be made.

There is growing interest in potential genetic contributions to tinnitus risk. Twin studies have estimated the heritability of tinnitus at 40–60% [60,61], although several candidate gene studies have failed to find such an association [62,63,64]. However, a recent (2020) large-scale, genome-wide association study (GWAS) in the UK Biobank and United States Million Veteran Program identified six genome-wide loci and 27 candidate genes associated with self-reported tinnitus among >170,000 people of European ancestry [65]. The estimated heritability was modest at 6%, but significant. This contrasts somewhat with the results of a prior GWAS that did not identify any significant candidate genes and estimated a lower heritability of 3.2%, which could be attributed to its comparatively much smaller population (*n* = 167) [66]. 

## 3. Current Options for Tinnitus 

Broadly, treatment mechanisms for bothersome chronic tinnitus can be subdivided into two categories: tinnitus perception and response to tinnitus. Treatments modulating tinnitus perception, such as electrical and magnetic stimulation, aim to reduce or eliminate symptoms. On the other hand, tinnitus-response treatments aim to reduce the patient’s negative affect or response to tinnitus and include cognitive behavioral therapy (CBT) and sound therapy.

**Figure 3:**
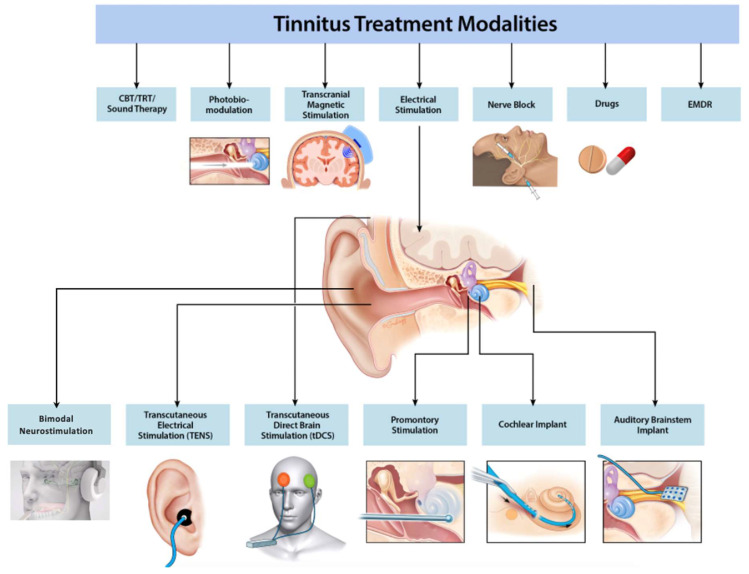
**Schematic of current and emerging tinnitus treatments.** Current and emerging treatments for tinnitus include CBT/TRT/sound therapy, transcranial magnetic stimulation (TMS), electrical stimulation (transcutaneous, promontory, cochlear implant, auditory brainstem implant), bimodal stimulation (Lenire^®^ device from Neuromod Devices [70,71]), nerve blocks, drugs, and EMDR.

The clinical guidelines for tinnitus from the AAO-HNF recommend hearing aid evaluation and CBT as options for chronic bothersome tinnitus and present sound therapy as another potential option [67]. The guidelines discourage the use of any medical drug therapy, dietary supplements, or repetitive transcranial magnetic stimulation (rTMS), given the lack of effective data at the time of the guidelines’ publication in 2014. More recent guidelines from Europe (2019) and Japan (2020) continue to strongly recommend CBT, but provide poor to no recommendations against dietary supplements, sound therapy, medications, rTMS, and supplements [68,69]. New advances in both medical and surgical modalities for tinnitus have been developed which may hold promise for treating this chronic condition (Figure 3). 

### 3.1. CBT

CBT, a type of psychotherapy, is used as an intervention for a wide variety of psychiatric conditions, including anxiety, depression, and the distress associated with tinnitus [72]. It aims to modulate negative thoughts associated with maladaptive behavior through reframing, using techniques like the development of positive coping skills, distraction, and relaxation. Duration of therapy for tinnitus can range from 8 to 24 weekly sessions with a trained professional [73].

To date, CBT is the only intervention for tinnitus to receive strong recommendations in clinical practice guidelines, but the benefits are primarily limited to managing tinnitus-related distress [67,68,69]. For example, several systemic reviews and meta-analyses have demonstrated that CBT is effective in improving patients’ negative interpretations of tinnitus, but that its impact on anxiety or health-related quality of life may be less than that of audiological care, and that evidence of long-term outcomes are lacking [74,75,76]. A recent (2020) Cochrane review found that, compared to waiting or receiving no treatment for tinnitus, CBT meaningfully improved perception of tinnitus severity (THI score) and, to a lesser extent, measures of quality of life, anxiety, and depression [74]. Similarly, CBT provided a greater improvement on quality of life compared to usual audiological care and tinnitus retraining therapy, but there was no difference seen in depression and anxiety given the lack of long-term follow-up [74]. A randomized controlled trial (RCT) of internet-based versus in-person CBT for patients with distressing tinnitus found that both modalities were equally effective in reducing tinnitus-related distress measured with the TFI [77], although a meta-analysis of RCTs indicated that in-person CBT was more effective for tinnitus-related quality of life [75]. However, internet-based therapies may offer access to therapy for a larger subpopulation of tinnitus patients, especially in the era of the COVID pandemic. Other forms of psychotherapy have also demonstrated beneficial effects for tinnitus. For example, an RCT comparing the efficacy of mindfulness-based cognitive therapy (MBCT) and intensive relaxation therapy for chronic, distressing tinnitus found significantly greater reductions in self-reported tinnitus severity with MBCT, and persistent effects at 6 months of follow-up [78]. Improvement was observed in both tinnitus loudness and severity, along with improvements in psychological distress. 

### 3.2. Hearing Aids

As the primary risk factor for tinnitus development is hearing loss, hearing aids are also recommended for chronic tinnitus [19]. This relationship however is not linear and many people with tinnitus have audiometrically normal hearing while many with severe hearing loss do not report tinnitus19. While hearing aids are recommended for patients with hearing loss and concurrent tinnitus in all guidelines [67,68,69], hearing aids for tinnitus alone is given a weak recommendation due to the lack of high-quality, robust data in the literature [69]. Several systemic reviews investigating the efficacy of hearing aids for tinnitus have found a lack of high-quality RCTs in the literature and noted equivocal results with a need for further studies [79,80]. 

### 3.3. Sound Therapy

Sound therapy aims to reduce the intensity of tinnitus by using an external sound to distract the listener [81]. This method is hypothesized to promote the habituation of tinnitus and stimulate the hypoactive neural auditory pathways impacted by hearing loss [38]. Therapy can be offered in the form of a device providing broadband low-level white noise or noise at the tinnitus frequency, or through a hearing aid to amplify external noise.

Numerous studies have investigated the efficacy of sound masking for managing tinnitus, although the results have been heterogenous. A Cochrane review investigating efficacy of the masking determined that there was weak evidence to show efficacy of sound therapy for tinnitus due to limited data and bias in the studies [82]. An RCT assessing the impact of masking, retraining therapy, educational counseling with hearing aids, or waiting (no intervention) on the perception of tinnitus severity reported similar improvement in all three intervention groups at 6 and 18 months, but no improvement in patients who waited [83].

### 3.4. Eye-Movement Desensitization Reprocessing 

EMDR is a form of conditioning psychotherapy traditionally used for post-traumatic stress disorder and more recently applied to tinnitus, with the most recent study published in 2018 [84]. EMDR is hypothesized to reduce tinnitus distress via desensitization and reprocessing of memories and images associated with negative perceptions of tinnitus [85]. As a newer form of therapy, few studies have investigated the efficacy of EMDR for the reduction of tinnitus-related distress, although initial reports have been positive [85,86,87]. Prospective trials on EMDR for chronic tinnitus have found clinically significant benefits on quality-of-life tinnitus surveys [85,86]. Further, an RCT comparing bimodal therapy with tinnitus retraining therapy plus EMDR or tinnitus retraining therapy plus CBT found that both treatment modalities resulted in equivalent reductions in tinnitus severity [87].

### 3.5. Cochlear Implantation

Cochlear implants are surgically implanted hearing prosthetics which electrically stimulate the auditory nerve and are therapeutic options for patients with moderate to severe hearing loss, with or without tinnitus, which has not improved with hearing aids. An estimated 68–86% of adult cochlear implant candidates also experience tinnitus [88,89]. Notably, numerous studies have reported secondary improvement or even resolution of tinnitus symptoms following cochlear implantation, ranging from 34 to 92% of implantees [90,91,92,93]. Systematic reviews and meta-analyses have similarly described the beneficial effects of cochlear implantation on both quality of life and tinnitus symptoms, with Levy et al. concluding that approximately 75% of implantees across 17 studies experienced improvement in tinnitus symptoms while 15% achieved complete resolution [94,95]. The exact therapeutic mechanism is unclear, but it has been hypothesized that increased afferent input to the auditory nerve from the implant may attenuate maladaptive neural activity and initiate neuroplastic reorganization of the central auditory pathways and associated brain areas [96]. This is supported by studies reporting gradual improvement in tinnitus symptoms over the course of several months post-implantation [88].

Interestingly, however, several studies have reported worsening of tinnitus severity in a subset of patients (<5%) following cochlear implantation [95,97]. Some cochlear implantation surgeries involve the creation of a cochleostomy for electrode insertion, and this trauma may damage remaining hair cells, potentially decreasing residual hearing and worsening tinnitus. However, modification of the surgical technique to a less traumatic round window approach to insert the electrode may reduce this trauma and associated risk [98]. For example, a prospective study by Kloostra et al. assessing the post-cochlear implantation outcomes of 44 patients (66% with preoperative tinnitus) who received either a cochleostomy or round window approach found that 75% who achieved tinnitus cessation received the round window approach versus cochleostomy [99]. Thus, it is possible that a round window approach instead of a separate cochleostomy for electrode insertion may offer advantages for patients with preoperative tinnitus, although further prospective studies are needed.

Additionally, there is some evidence that the duration and chronicity of tinnitus prior to implantation impacts the likelihood of tinnitus cessation, perhaps due to entrenchment of maladaptive changes [99,100,101]. The abovementioned study by Kloostra et al. noted that the mean time from tinnitus onset was 32.2 years for patients who did not have cessation of tinnitus symptoms versus just 5.2 years for patients who achieved cessation [99]. Similarly, Miyamoto et al. observed that preoperative tinnitus duration of <20 years was significantly correlated with post-implantation improvement in tinnitus symptoms among 78 adult implantees [102]. While cochlear implantation remains a viable option for the treatment of tinnitus with accompanying hearing loss, there is a risk of worsening tinnitus, and thus should be used for patients who specifically want to improve their hearing ability.

## 4. Emerging Options for Tinnitus 

### 4.1. Electrical Stimulation

Electrical stimulation can be divided broadly into three categories: direct cutaneous stimulation (DCT), inner ear stimulation (promontory stimulation and cochlear implants), and intraneural implants.

#### 4.1.1. Transcranial and Transcutaneous Stimulation

Non-invasive electrical stimulation techniques to modulate neural hyperactivity in specific brain areas include transcutaneous direct current stimulation (tDCS) and transcutaneous electrical stimulation (TENS) [103]. Neuroimaging studies have reported structural abnormalities and hyperactivity in the left auditory cortex/temporoparietal region, dorsolateral prefrontal cortex (DLPFC), and limbic system associated with tinnitus perception or distress; thus, modulation of activity is primarily directed to these regions [104,105,106,107,108].

In studies of tDCS for tinnitus, stimulation intensity varies (i.e., 2–15 mA) but electrodes are generally placed over the temporoparietal region for several consecutive sessions. In a 2020 double-blind, placebo-controlled RCT, 24 patients with chronic tinnitus received five consecutive sessions of either tDCS (2 mA for 20 min) or a sham stimulation (*n* = 12 each) to the left temporoparietal region and right DLPFC [109]. The tDCS-treated patients reported significant improvements from baseline in tinnitus annoyance and severity (i.e., Tinnitus Handicap Index (THI)/Visual Analogue Scale (VAS)) and decreased alpha/beta/theta frequency, as measured by electroencephalogram, immediately following intervention. Additionally, reduced electrical activity was observed in the frontal, temporoparietal, and limbic regions of tDCS-treated patients. However, several prior RCTs and prospective studies of tDCS in patients with chronic tinnitus failed to demonstrate similarly significant effects [110,111,112], and a scoping review noted the transient nature of any benefit [113].

TENS involves cutaneous electrode placement, typically over the auricle or around the mastoid to target the auricular branch of the vagus nerve, or is delivered via a probe in the external auditory canal. A placebo-controlled, randomized prospective study of unilateral (*n* = 20) or bilateral (*n* = 20) cutaneous TENS for chronic tinnitus reported that scores on the THI and a survey of depression significantly improved in both TENS-treated groups compared to their pre-intervention baseline [114]. However, a significant placebo effect was observed among the sham-treated patients as well. Another RCT of cutaneous TENS for chronic tinnitus reported no significant benefit for the active intervention group as well as large placebo effects among sham-treated patients [115]. In a large study of 500 patients with tinnitus who received probe TENS to the tympanic membrane, approximately half (53%) reported some benefit in symptom reduction and 7% had complete suppression of tinnitus [116]. Placebo effects were not assessed in that study, 13 patients experienced worsening of tinnitus, and 27% of the patients who reported initial benefits reported no benefit at 3 months. 

Taken together, the evidence suggests that TENS may confer transient beneficial effects for tinnitus, but may be due to a high placebo effect and have no obvious long-term benefit. Three recent systematic reviews and meta-analyses likewise concluded that that electrical stimulation may indeed provide a benefit for tinnitus, but high bias is present in most studies and further investigation into the most effective stimulation pattern and modality are needed before recommendation to patients [117,118,119].

#### 4.1.2. Promontory and Round Window Stimulation

Inner ear stimulation techniques include promontory and round window biphasic stimulation [120,121,122]. Prospective studies assessing trans-tympanic promontory probe stimulation among small cohorts have demonstrated some significant improvements in tinnitus symptoms during or soon after intervention, but a return to baseline and loss of benefit at longer-term follow-up when stimulation is stopped. For example, in a study of ten patients with severe unilateral tinnitus who received multiple consecutive trans-tympanic needle electrode stimulation pulses to the promontory, self-reported tinnitus loudness (assessed with the VAS) and THI scores significantly improved for five patients, but VAS returned to baseline 4 weeks post-intervention [120]. Additionally, there were no changes in measures of tinnitus-specific audiological tests (i.e., minimum masking level, tinnitus loudness, and pitch) from the baseline. A small study by Wenzel et al. repurposed a cochlear implant to provide long-term (4 h per day for 3.5 years) stimulation via a non-penetrating ball electrode to the round window of patients with tinnitus and unilateral deafness (*n* = 3) [121]. At least one measure of tinnitus severity improved for all patients, although the electrode was eventually removed when the patients received conventional cochlear implants, the outcomes of which were not compared. There have also been recent efforts to develop an implantable system for suppression of tinnitus symptoms, with one ongoing open-label clinical trial testing feasibility in 16 adults with disruptive, intractable chronic tinnitus [123].

### 4.2. Repetitive Transcranial Magnetic Stimulation

Repetitive transcranial magnetic stimulation (rTMS) uses an electromagnetic field to noninvasively stimulate neural networks and modify dysfunctional cortical networks from outside the cranium [124]. Repetitive application of the magnetic field is proposed to induce lasting changes in targeted cortical regions and associated networks, and is routinely used for conditions such as refractory depression and obsessive–compulsive disorder [124]. Currently, it is also experimentally used for chronic tinnitus. While there is no consensus on the correct protocol for rTMS, it typically involves the placement of an electromagnetic coil over the left temporoparietal junction (TPJ), auditory cortex, or multiple sites that emits at low frequency (1 Hz) and 100−110% of resting motor threshold [113,125,126,127]. Participants may undergo resting state functional connectivity MRI prior to and immediately following rTMS treatment to assess structural or connectivity changes. The most common side effect of rTMS is a transient headache, but can rarely lead to a generalized seizure [128].

There is not yet consensus in the literature on the efficacy of rTMS for chronic tinnitus. In 2015, a double-blind RCT of rTMS applied to the TPJ of patients with chronic, bothersome tinnitus reported no significant functional connectivity changes or improvement from baseline THI score in those who received active (*n* = 16) or sham therapy (*n* = 14) [127]. The authors concluded that the TPJ alone may not be the ideal target for rTMS therapy for tinnitus, and successful neuromodulation may require multiple or personalized stimulation targets. Other studies have also investigated the ideal location(s) for rTMS stimulation for tinnitus. A randomized study by Khedr et al. compared the effect of rTMS delivered to the TPJ contralateral or ipsilateral to tinnitus in 62 patients with unilateral chronic tinnitus [129]. The results indicated that significantly greater improvement in THI score was achieved with contralateral stimulation than either ipsilateral or left side stimulation at 10 months, with no effect from frequency type (1 and 25 Hz), although there was no sham therapy group in that study. However, a similar RCT by Kim et al. assessing 1 Hz rTMS at the TPJ contralateral or ipsilateral to unilateral tinnitus did not find any differences between stimulation laterality, as both modalities resulted in significant improvements in THI and VAS scores at 1 month [130]. 

Further improvement and reliability in responses may be seen with personalized, targeted stimulation targets, as performed for depression [131]. Recent efforts from Lan et al. assessed neuroimaging indicators for optimal chronic tinnitus treatment [132]. Functional neural connections were assessed with resting state functional MRI, with preliminary results suggesting that patients with neural connections in the salience network–right frontoparietal network may respond better to rTMS [132]. However, further robust investigation is needed before rTMS can be formally recommended for chronic tinnitus.

### 4.3. Nerve Block

Nerve blocks have also been investigated for non-somatosensory tinnitus. A retrospective chart review assessed auriculotemporal nerve and facial nerve blocks with lidocaine in 55 patients with chronic (>6 months, *n* = 40) or sub-acute (>3 months, *n* = 15) tinnitus after trigeminal and facial nerve stimulation and other treatments (i.e., intratympanic steroids or medications) [133]. The results indicated that approximately 88% of patients experienced some improvement in scores on a modified VAS after several integrative treatments (stimulation and block). Transient facial palsy (5–15 min) was noted right after the nerve block, which spontaneously resolved.

Other nerve blocks in the literature have targeted the occipital nerve to reduce tinnitus-associated otalgia [134,135]. A retrospective study of 33 patients with tinnitus and otalgia underwent ultrasound guided occipital nerve blocks, with significant reduction in immediately after the nerve block [134]. Long-term results, however, were varied and patients in this study had several varying etiologies for otalgia including somatosensory tinnitus (temporomandibular joint pain, myofascial pain syndrome, and cervical stenosis) which limits generalizability. Further investigations and dedicated studies are needed to elucidate the benefit of nerve blocks for chronic tinnitus.

### 4.4. Bimodal Neuromodulation

Bimodal neuromodulation, which pairs sound and electrical stimulation of peripheral nerves, is an emerging therapy for tinnitus. Bimodal neuromodulation is thought to drive plasticity and changes in the auditory pathway (midbrain, cortex, or brainstem) involved with tinnitus in several animal studies [136,137]. Congruent with animal studies, clinical trials have also appeared promising for chronic tinnitus, with the Food and Drug Administration (FDA) granting de novo approval for the biomodulation wearable Lenire^®^ in March of 2023 [138]. Lenire^®^ is a Class IIa device delivering electrical stimulation to the tongue with an oral device and sound stimulation. In a randomized, blinded trial of 326 patients with chronic subjective tinnitus, the efficacy of Lenire^®^ was tested in three separate groups with different stimulation settings. Over a 12-week period, all intervention groups had a statistically significant reduction in tinnitus symptom severity, with sustained therapeutic improvement seen at a 12-month follow-up [71]. However, there were diminishing returns on the second 6 weeks of treatment, likely due to treatment habituation. In a follow-up clinical trial, there were enhanced therapeutic benefits in tinnitus symptom severity achieved by changing stimulation parameters during the second 6-week treatment period, overcoming treatment habituation [70]. Bimodal stimulation appears promising for the treatment of chronic tinnitus, with the advantage of an FDA-approved at-home device for treatment. Further investigations will be needed to follow up long-term therapeutic benefit past 12 months, to delineate specific stimulation patterns among tinnitus populations, and to evaluate the effect of bimodal stimulation over a placebo in a real-world setting. 

### 4.5. Pharmaceutical Therapy

Psychiatric comorbidities associated with tinnitus include depression, anxiety, and obsessive–compulsive disorder traits [26,139]. Accordingly, antidepressants and antipsychotics for chronic tinnitus have been assessed in several RCTs, but have failed to show evidence of efficacy while also reporting common adverse events with these drugs [140]. A double-blind, placebo-controlled study of trazadone for bothersome tinnitus (<1 year duration and normal audiograms) found that patients receiving trazadone (*n* = 43) experienced similar improvements in tinnitus severity, quality of life, or tinnitus-related discomfort as those receiving a placebo (*n* = 42) [141]. Similarly, an RCT of 115 non-depressed patients with chronic tinnitus did not find any significant benefit with the selective serotonin reuptake inhibitor paroxetine compared with a placebo [142]. A 2012 Cochrane review concluded there was insufficient evidence of the efficacy of antidepressants for tinnitus due to poorly randomized and low-quality studies, and further investigation was warranted [140].

Lidocaine interferes with fast-gated sodium channels and is hypothesized to inhibit hypersensitivity in the central auditory pathways [143]. Accordingly, lidocaine has been investigated as a pharmaceutical option for tinnitus, delivered either intravenously or trans-tympanically, although most beneficial effects at tinnitus suppression appear to be transient [144,145,146,147]. A double-blind, cross-over study by Baguley et al. assessed the inhibitory effects of intravenous 2% lidocaine (1.5 mg/kg) versus saline placebo in 16 patients with postoperative tinnitus following translabyrinthine resection of unilateral, sporadic vestibular schwannoma [148]. Interestingly, patients who received lidocaine had significant improvements in tinnitus loudness and distress (measured with VAS) compared to placebo patients at 5 min, but not at 20 min, post-infusion. Importantly, intravenous lidocaine is associated with several adverse events, including the risk of increased tinnitus severity. For example, in an 1983 double-blind RCT, over 30% of tinnitus patients treated with intravenous lidocaine (100 mg) reported worsened sensation of tinnitus, and there was a high rate of adverse events (e.g., disequilibrium, slurred speech, numbness, and tingling of the extremities) [146]. Subsequent small cohort studies have investigated intratympanic lidocaine injections for subjective tinnitus, with heterogenous results [149,150,151]. However, the AAO-HNF tinnitus treatment guidelines do not recommend intratympanic lidocaine injections due to the lack of strong efficacy evidence, or intravenous lidocaine due to the risk of adverse events [67].

Benzodiazepines have also been investigated in several RCTs but lack robust literature to support their use. Benzodiazepines are hypothesized to bind to GABA receptors and enhance inhibitory signals, thus modulating hyperactivity signals associated with tinnitus [152]. A randomized cross-over trial by Han et al. compared the effects of clonazepam and Ginkgo biloba [153]. There was significant improvement in tinnitus loudness, duration, and annoyance in the clonazepam group, but no significant differences in the Ginkgo biloba group. Accordingly, several prior systemic reviews have found no benefit in the use of Ginkgo biloba for the treatment of chronic tinnitus [154,155]. The efficacy of alprazolam, on the other hand, has been equivocal with conflicting results in the literature [156,157]. Given the lack of robust literature and side effect profile of benzodiazepines, benzodiazepines are not recommended for the treatment of subjective tinnitus alone at this time [67,69].

## 5. Other Limited Evidence Treatments

Photobiomodulation (PBM) or low-level laser therapy (LLLT) is an investigative treatment that utilizes low power light to modulate neural activity. The therapeutic mechanism of PBM is yet unclear but is proposed to be cellular-level stimulation of cytochrome C, synthesis of growth factors, and activation of repair mechanisms in the inner ear [158]. PBM tinnitus treatment involves the placement of a trans-meatal probe to delivery laser therapy, generally in the visible or near-infrared spectrum (532–1064 nm), at varying power levels (5–100 mW) [158]. Previous RCTs and studies of the efficacy of PBM for tinnitus have reported conflicting results [159,160,161,162,163,164]. For example, a double-blind RCT of trans-meatal LLLT (810 nm at 60 mW) for chronic, disabling tinnitus found no significant differences in any measures of tinnitus between active treatment (*n* = 23) or placebo groups (*n* = 20) [165]. A large systematic review of RCTs of LLLT for tinnitus (2022) similarly found conflicting results regarding the benefit of PBM given the heterogeneity of study designs, high levels of bias, equivocal results, and lack of long-term follow-up [166].

Migraine medications have also been investigated for treatment of chronic tinnitus. The disruption of somatosensory and auditory inputs of the trigeminal nerve have been implicated in the pathophysiology of tinnitus as the dorsal cochlear nucleus receives indirect input from the trigeminal nerve [167]. This suggests a potential connection between tinnitus and migraines [168]. Many migraine patients have auditory manifestations, including tinnitus. Thus, migraine medications may serve as an option to potentially reduce the severity of tinnitus in a subset of patients, but further robust trials are warranted at this time. One active clinical trial aims to assess the effects of nortriptyline/topiramate and verapamil/paroxetine migraine medications in reducing the severity of tinnitus [169]. 

## 6. Conclusions and Future Directions

Over the past several decades, advances in basic and clinical research have greatly improved our understanding of tinnitus. Despite these advances, the overall impact of available tinnitus treatment remains poor without consensus, and is a source of frustration for patients. In a 2018 survey of patients regarding their expectations and the outcomes of tinnitus treatment, 36% responded with having “no expectation” and 49% reported that their treatment was “not at all successful” [170]. The lack of both diagnostic and definitive treatment options remains a huge challenge in the management of chronic subjective tinnitus. The most common treatments, such as hearing aids, CBT, and masking, address patients’ response to tinnitus, but viable treatment modalities for tinnitus suppression remain few. 

Tinnitus research remains challenging owing to a lack of methodological standardization in both research and clinical trials, as well the need for longer-term follow-up of patients in existing trials [171]. There is a lack of strong objective measures, and a strong placebo effect present in many tinnitus trials [172]. Many validated tinnitus questionnaires exist and have favorable psychometric signatures, and thus there is no consensus or standardization for a specific questionnaire in either research or clinical use [173,174,175]. This lack of standardization in questionnaires remains a challenge for making accurate comparisons between treatment or research groups. There have also been recent efforts to distinguish tinnitus as a symptom and tinnitus as a disorder. Tinnitus disorder specifically reflects the associated psychological and physical impacts of the disease [15]. Standardization of these processes will prove paramount for creating a lasting impact on future tinnitus research

However, the future of tinnitus research remains bright as new technologies are rapidly emerging. In recent years, several candidate genes for tinnitus have been identified, as well as plasma metabolomic biomarkers of persistent tinnitus, which may open up exciting new avenues for diagnostics, gene-based therapeutics, and alterative therapy development [65,176]. A recent (2022) metabolomic study that compared the blood plasma levels of 466 metabolites between women with persistent tinnitus (*n* = 488) and controls without tinnitus (*n* = 5989) identified several novel biomarkers positively or inversely associated with chronic tinnitus [177]. Compounds such as triglycerides and diglycerides were positively associated with tinnitus, while other cholesterol metabolites such as cholesteryl esters and lysophosphatidylcholines were inversely associated with tinnitus. While the precise roles of these compounds in tinnitus require further investigation, lipid dysregulation may play a role in tinnitus pathogenesis, consistent with other neurodegenerative disorders such as Alzheimer’s and Parkinson’s disease [178,179]. Additionally, a recent study by Amanat et al. assessing genetic risk factors identified several rare synaptic genes in patients with severe tinnitus, and the authors were able to replicate these findings between two European cohorts (Spanish patients with Meniere’s disease and Swedish tinnitus patients) [180]. These rare synaptic genes associated with membrane trafficking and cytoskeletal protein binding were replicated between these cohorts irrespective of their underlying hearing disorder, demonstrating the possible effect of rare variants in severe tinnitus.

Furthermore, as of November 2022, 50 clinical trials of treatments for tinnitus are registered at ClinicalTrials.gov as currently recruiting (33 of 50) or preparing to recruit. One ongoing clinical trial is enrolling patients with incapacitating unilateral tinnitus to determine the efficacy of auditory brainstem implants [181], devices originally developed for people with hearing loss due to non-functional cochlear nerves. The early results of this therapeutic application are encouraging, as a retrospective study of patients with neurofibromatosis type 2 who received auditory brainstem implants demonstrated tinnitus suppression and reduction in tinnitus severity [182]. Additionally, vagal nerve stimulator implants and deep brain stimulation are being actively investigated. Data are limited at this time, and further investigations will be needed to delineate their effects on tinnitus [183,184]. Overall, interdisciplinary research linking the genetic, diagnostic, and therapeutic modalities will be critical to develop an effective and reproducible cure for chronic subjective tinnitus.
